# Implementing digital sexual and reproductive health care services in youth clinics: a qualitative study on perceived barriers and facilitators among midwives in Stockholm, Sweden

**DOI:** 10.1186/s12913-024-10932-1

**Published:** 2024-04-02

**Authors:** Linn Zettergren, Elin C. Larsson, Lovisa Hellsten, Kyriaki Kosidou, Anna Maria Nielsen

**Affiliations:** 1 Department of Global Public Health, Karolinska Institutet, Tomtebodavägen 18a, Widerströmska Huset 171 77, Stockholm, Sweden; 2 Center for Epidemiology and Community Medicine, Region Stockholm, SE-104 31 Stockholm, Box 45436, Sweden; 3 Department of Womens and Childrens Health, Tomtebodavägen 18a, Widerströmska Huset, 171 77 Stockholm, Sweden

**Keywords:** Youth clinics, Implementation research, Midwives, Health care providers, Digital health, Telehealth, CFIR, Sexual and reproductive health, Adolescent, Young adults, Health services research, Sweden

## Abstract

**Background:**

Digital health care services have the potential to improve access to sexual and reproductive health care for youth but require substantial implementation efforts to translate into individual and public health gains. Health care providers are influential both regarding implementation and utilization of the services, and hence, their perceptions of digital health care services and the implementation process are essential to identify and address. The aim of this study was to explore midwives’ perception of digital sexual and reproductive health care services for youth, and to identify perceived barriers and facilitators of the implementation of digital health care provision in youth clinics.

**Methods:**

We performed semi-structured interviews with midwives (*n* = 16) working at youth clinics providing both on-site and digital sexual and reproductive health care services to youth in Stockholm, Sweden. Interview data were analyzed using a content analysis approach guided by the Consolidated Framework for Implementation Research (CFIR).

**Results:**

Midwives acknowledged that the implementation of digital health care improved the overall access and timeliness of the services at youth clinics. The ability to accommodate the needs of youth regarding their preferred meeting environment (digital or on-site) and easy access to follow-up consultations were identified as benefits of digital health care. Challenges to provide digital health care included communication barriers, privacy and confidentiality concerns, time constraints, inability to offer digital appointments for social counselling, and midwives’ preference for in person consultations. Experiencing organizational support during the implementation was appreciated but varied between the respondents.

**Conclusion:**

Digital sexual and reproductive health care services could increase access and are valuable complements to on-site services in youth clinics. Sufficient training for midwives and organizational support are crucial to ensure high quality health care. Privacy and safety concerns for the youth might aggravate implementation of digital health care. Future research could focus on equitable access and youth’ perceptions of digital health care services for sexual and reproductive health.

**Supplementary Information:**

The online version contains supplementary material available at 10.1186/s12913-024-10932-1.

## Background

Ensuring access to appropriate and acceptable health care services is fundamental for the realization of sexual and reproductive health and rights (SRHR) for all individuals [1, 2]. The need for comprehensive and individually tailored support for sexual and reproductive health is of particular importance throughout adolescence and young adulthood, a period characterized by major transitions such as puberty onset, sexual exploration and lifestyle changes [1]. On a global level, much has been achieved in the field of young people’s SRHR, such as provision of comprehensive sexual education and accessibility to youth friendly clinics. However, unmet needs are still prevalent with inequities both in terms of access *to*, and utilization *of* sexual and reproductive health care services, though challenges may vary between different contexts [1, 3]. Globally, challenges of accessing these services include limited financial resources and means of transportation. In Sweden and elsewhere, health seeking behavior is often affected by social stigma surrounding youth sexuality and concerns regarding confidentiality and privacy in the health care setting, and uptake of services can consequently be low [3–5].

Digital health care services, such as mobile or computer-based consultations with health care providers, offer novel modes of health care delivery with the potential to improve access to services as well as health outcomes [6, 7]. Given the widespread adoption of digital technology and high level of digital literacy among young people, the use of digital tools and interventions in health care has been particularly popular to target the youth population [8, 9]. Digital services however often place high demands on healthcare organizations and require substantial implementation efforts to translate into public health gains [6, 10].

In Sweden, digital health initiatives have been increasingly promoted following the adoption of a national eHealth strategy in 2016 [11], and the rapid upscaling of digital solutions in the wake of the Covid-19 pandemic. Digital technology is widely available and used by the Swedish population: among 16–24-year-olds 97% have internet at home and 95% are using it multiple times a day [11]. In this setting, digital health care services have the potential to improve service accessibility while providing a safe communication channel between health care providers and youth. One example of this is the implementation of digital services, i.e. the introduction of video consultations and a chat function, in youth clinics for sexual and reproductive health care in Region Stockholm, where this study is set.

The first youth clinic in Sweden was launched in 1970, there are approximately 220 clinics in the country, and 31 within Stockholm County. Youth clinics offer free of charge services to young people aged 12–23 (or 12–25 in some Counties) with emphasis on SRHR and psychosocial support. Services provided include SRHR education, testing for sexually transmitted infections, contraception counselling, and mental health support. Youth clinics strive to promote youth’ personal development, prevent illness and to provide support for psychological and social issues. The staff comprise of midwives, behavioural therapists, social counsellors and physicians [12]. Swedish midwives have an independent role within healthcare related to SRHR [13]. In addition to the youth clinics, the national platform for youth clinics on-line (umo.se) has since 2008 served as a knowledge centre for youth regarding e.g. physical development, sexual relationships and mental health [14].

The success and sustainability of digital interventions and services involves various stakeholders including health care providers and caretakers [15]. Implementation can consequently be both impeded and facilitated by determinants across multiple levels [15]. Systematic reviews and other research, conducted foremost in high-income settings, have highlighted several challenges related to implementation of digital health care services across various health care fields including sexual and reproductive health [15–21]. Challenges include difficulties in technology-use, resistance to change among health care providers, reimbursement issues, workflow changes, and concerns regarding the confidentiality and privacy of patients [15–21]. Additionally, intrapersonal factors, such as the ability to establish trusting relationship with patients through digital services, changes in communication dynamics and loss of non-verbal cues, have been identified as potential negative outcomes of digital service delivery [15–21]. Facilitators to uptake of digital services among health care providers included the recognition of patient benefits, having received training, fit of the digital services with existing workflows, and flexibility in how, and from where, services can be delivered [16, 17]. Consequently, health care providers can in turn affect implementation outcomes such as acceptability, adoption, and appropriateness of digital services [22].

To our knowledge, no prior evaluation has been conducted at the youth clinics for sexual and reproductive health to explore how digital services are perceived by health care providers, i.e. midwives. There is a lack of knowledge pertaining to the experiences of midwives involved in digital sexual and reproductive health care provision targeting youth in Sweden and similar high-income settings. The aim of this study was to explore midwives’ perceptions of digital sexual and reproductive health care services for youth, and to identify perceived barriers and facilitators of the implementation of digital health care provision in youth clinics in Stockholm, Sweden.

## Methods

### Theoretical framework

This study draws on the Consolidated Framework for Implementation Research (CFIR) to identify determinants either facilitating or hindering successful implementation of digital sexual and reproductive health care services in youth clinics. CFIR is a comprehensive determinant framework providing a pragmatic structure to explore and evaluate multi-level determinants of implementation success of an intervention in a given context [23]. It comprises 39 constructs across five domains: (1) intervention characteristics (the digital health care services), (2) outer setting (the health care system), (3) inner setting (the youth clinics organization), (4) characteristics of involved individuals (health care providers/midwives), and (5) implementation process (strategies to implement the intervention) (Appendix 1). In this study, the framework was used to guide both data collection and analysis of midwife’s perceptions of digital sexual and reproductive health care services in youth clinics.

### Study setting

The study was carried out in youth clinics in Region Stockholm, Sweden in 2022, which serve approximately 300 000 young people aged 12–22 annually, hereafter referred to as “youth” [24]. As of 2021 the publicly funded Stockholm Region Health Care Services managed 29 out of 31 on-site youth clinics as well as a recently implemented region-wide online clinic. Youth clinic staff mainly consists of midwives and social counselors. Midwives and counselors are however employed by different organizations, i.e. the management of the youth clinics are divided between the municipality and the county council (the Region Stockholm).Social counselors were not involved in provision of care through digital services and were therefore excluded from this study. The study population in this study constitutes midwives working at the youth clinics in Region Stockholm.

### Digitalization in youth clinics

In 2018, digital consultations with midwives at youth clinics in Stockholm were initially piloted in one of the clinics. It has since developed into a region-wide online clinic alongside the introduction of digital services at all 29 on-site youth clinics. The digital intervention comprises consultations with midwives employed at the youth clinic, either through video or using an asynchronous chat function via a digital health care application (*“Always Open”, “Alltid Öppet”*). In order to get access to the digital services, youth must use electronic identification, i.e. their personal identity number in combination with a code which is considered equivalent to a physical ID-card. The implementation of the online youth clinic was accelerated by the Covid-19 pandemic in 2020–2022 due to on-site service limitations and an increased demand for digital alternatives. The online youth clinic has its own staff, but the digital services are also integrated in the on-site clinics where midwives may chat or schedule digital appointments via video and follow-up sessions with youth. Additionally, each on-site clinic must offer a pre-set number of digital appointments per week. The total number of visits to the different clinics ranges from approximately 1400 per year to 8500 per year. The online clinic is one of the largest clinics within the organization with approximately 7300 visits per year (unpublished data from the Region Stockholm 2021–2022). An equal amount of time is allocated for the digital video consultations and the on-site consultations, i.e. 30 min, a guideline that applies to all clinics in the Region Stockholm.

### Sampling strategy

The study population consisted of approximately 120 midwives working in youth clinics in the Region Stockholm. A purposive sampling strategy following the principle of maximum variation was applied to capture potential differences in perceptions emerging from variation in clinic settings and individual characteristics [25]. Individual level characteristics that were considered included age and length of work experience, both as a midwife and specifically in the youth clinic setting. Clinic characteristics of interest were geographic location, size, and socioeconomic status of the area. Midwives had to be employed by the youth clinic and have experiences of both digital and on-site services to be eligible for participation. Initially 18 clinics, with a variation of geographical location (city, close-suburb, suburb) and socioeconomic status of the area were approached through the heads of the clinics, to explore interest of participating in the study. Out of 18 contacted clinics, 15 (including the online clinic) provided contact details to midwives meeting the eligibility criteria. The three clinics who did not respond to our request were each situated in a low-, a middle-, and a high socioeconomic area. Data was collected at two different time periods which enabled the purposive sampling strategy regarding the respondents’ characteristics, i.e. we reminded clinics to provide contact details to midwives and thus we were able to purposely include midwives of different ages and variation of working experiences (years) at the youth clinics.

### Sample

In total 16 midwives from 15 clinics took part in the study (Table 1). The majority (*n* = 14) of the respondents were partially working digitally and two were employed at the online clinic but had prior experience of working at an on-site clinic. Out of the 14 on-site clinics, three were situated in high socioeconomic areas, six were situated in middle socioeconomic areas, and the remaining five were situated in low socioeconomic areas with a high proportion of migrant population.

**Table 1:** Respondents and clinics characteristics

Respondents	*n* = 16
Age (years)	
Range	29–65
Mean	48
Time worked at clinic (years)	
Range	1–21
Mean	8
Gender	
Women	16
Men	0
Location of youth clinic	
Stockholm city	7
Greater Stockholm area	5
Stockholm County	2
Only online	2

### Data collection

Semi-structured interviews were conducted during two time periods, February-Mars (*n* = 8) and November-December (*n* = 8) in the year 2022. All interviews were conducted by the first author (LZ), the different data collection periods related to practical issues regarding LZ masters studies and her latter employment at the Karolinska Institutet. The interviews were either conducted in an enclosed office space at the clinic of the respondent (*n* = 12) or via video (*n* = 4) and ranged from 33 to 71 min (mean = 52 min)., using a structured interview guide based on CFIR and previous research (Appendix 2). The interview guide was internally tested and piloted during the first interview, resulting in some clarifications and changes to improve flow [26]. Questions were open-ended, and the interviewer consistently used probes to elicit illustrating examples and in-depth explanations as well as concluding reiterations to minimize risk of misinterpretations [27].

The questions focused on midwives´ perceptions on how digital consultations differ from on-site meetings, the digitalization’s effect on accessibility of SRHR services, and organisational support during the implementation of digital health care services. Before closing the interviews, respondents were given the opportunity to bring up additional topics or questions. Field notes were taken immediately after the interviews to reflect on the context, the researcher-respondent interaction, and initial reflections on the interview content [27, 28]. Field notes were also used to indicate data saturation by highlighting novel topics and perceptions from each interview. After 16 interviews, no new meaningful data emerged, and saturation was considered met [29]. All interviews were audio recorded and transcribed verbatim by LZ.

### Data analysis

Data were analyzed using a combined deductive and inductive approach to qualitative content analysis [30, 31]. This method allowed the CFIR framework to guide the analytical structure under the pre-defined CFIR-constructs (deductive approach), yet remain open to novel data-driven codes (inductive approach). The initial step of the analysis involved researchers immersing themselves in the data by repeatedly reading through transcripts and field notes. An unconstrained coding matrix based on the five CFIR-domains and the 39 constructs was then developed. The CFIR-constructs were included in the coding matrix along with the coding criteria for qualitative data analysis provided by the CFIR resource base (Appendix 1) [32, 33]. Coding was conducted using NVivo software in which interview data was analyzed for relevance to the research question and sorted according to the coding matrix [34]. Meaning units identified consisted of one or more sentences. If a meaning unit resonated with more than one CFIR-construct it was sorted under the most relevant construct after consulting with the research team. Two transcripts were initially coded separately by LZ and LH and then discussed to ensure the codebook was used consistently. Overall, the consistency was high, however some clarifications and examples were added to the coding criteria to assure credibility of the data. The remaining 14 transcripts were coded by LZ. Following the deductive coding process, data-driven codes were created for the meaning units for the CFIR-constructs in the coding matrix using inductive content analysis [30]. Based on the respondents perceptions, each code was considered either a barrier or a facilitator to the implementation of the digital health services. Barriers and facilitators were reviewed by the research group to ensure names accurately reflected their content and were clear to the reader. Lastly, for each domain, a data-driven theme reflecting its content was created.

### The research team

At the time of the study, the research team consisted of five female researchers, one masters student (LZ) who conducted the data sampling, data collection, and data analysis, one doctoral student (LH) who conducted the data analysis in parallel with LZ. Neither of the two had prior clinical experience from the youth clinic and thus, preconceptions and assumptions that could affect the analysis process were avoided. The three senior researchers (AN, EL, KK) conceptualized the study. While four out of five members of the research team were employed by, or affiliated with, the Region Stockholm, it was Karolinska Institutet who conducted the study.

## Results

In the initial deductive content analysis, all five domains of the CFIR framework and 21 out of the 39 CFIR-constructs were identified. Subsequently the data-driven inductive content analysis revealed that midwives’ positive attitudes regarding the digital services were considered implementation facilitators, and the negative comments were considered implementation barriers. In total, 41 barriers and facilitators were identified.

The themes developed based on the domains were: (1) Increased flexibility for the organization, midwives, and youth (The intervention characteristic domain). (2) Lowering the threshold for some youth, but efforts needed to ensure equitable access and utilization of services. (The outer setting domain). (3) Mixed experiences of organizational support and implications for quality of care. (The inner setting domain). (4) Midwives appreciate the digital consultation option but prefer meeting youth face to face. (The characteristics of individuals domain), and (5) The importance of knowledgeable and enthusiastic colleagues during the implementation process of the digital health care services (The process domain).

In Table 2 we present the themes, the identified constructs, and the barriers and facilitators related to implementation of digital health care services at the youth clinic.

**Table 2:** Themes based on Consolidated Framework for Implementation Research (CFIR) domains (1–5), CFIR constructs, and perceived barriers and facilitators related to implementation of digital health care services

1. Digital health services: Increased flexibility for organization, midwives and youth. (The intervention characteristic domain)		
*CFIR constructs*	*Facilitators*	*Barriers*
Relative Advantage	Improved flexibility for midwives and youth clinics. Timely youth-centered services. Simplifying patient-provider continuity*	
Complexity	Easy-to-use software	
Design Quality & Packaging	Useful digital tools and information in software	Insufficient documentation structure for digital meetings Short digital appointment timeframe*
*2. The health care system*: Lowering the threshold for some youth, but efforts needed to ensure equitable access and utilization of services. *(The outer setting domain)*		
*CFIR constructs*	*Facilitators*	*Barriers*
Patient Needs & Resources	Ability to meet diversity in needs. Improved access for hard-to-reach groups*	Inequities in digital access for youth* Lacking input and feedback of digital services from youth Exacerbating language and age-related barriers in digital meetings
Peer-pressure	Competing services from private digital caregivers	
External policy & Incentives	Accelerated digital availability due to Covid-19 pandemic response measures.	
*3. The organization at the youth clinics: Mixed experiences of organizational support and digital implications on quality of care. (The inner setting domain)*		
*CFIR constructs*	*Facilitators*	*Barriers*
Structural Characteristics	Region-wide collaboration and unity	Divided management undermines digital interprofessional collaboration.
Networks & Communications	Open organizational communication climate	
Tension for Change	Digitalization being ‘a sign of the times.	
Compatibility	Fits with workflows and tasks* Health examination appropriateness*	Privacy and safety concerns* Inadequate for a holistic health approach Loss of non-verbal cues and conversational depth
Relative Priority		Competing organizational priorities
Goals & Feedback	Feedback opportunities and receptiveness*	Goals of digital services remain undefined. Concern over future directions
Leadership Engagement		
Available Resources	Multilevel leadership support*	
	IT-support and digital infrastructure*	
Access to Knowledge & Information	Available guidelines/instructions Introduction and training opportunities*	
*4.The health care providers: Midwives appreciate the digital option but prefer meeting youth face to face. (The characteristics of individuals domain)*		
*CFIR constructs*	*Facilitators*	*Barriers*
Knowledge & Beliefs about the intervention	Overall belief in the health benefits of digital service options	Midwives´ meeting preferences* Habit of clinic-based work Youths’ expectations on the digital meeting
Self-efficacy	Increased digital confidence -practice makes perfect. Overcoming initial digital concern	Clinic setting offers more resources and higher confidence among midwives.
Individual State of Change	An intent to suggest digital options for youths more often.	
Other personal characteristics	Digital meetings require prior youth clinic experience of health care providers	
*5. Implementation strategies of the digital health services: The importance of knowledgeable and enthusiastic colleagues during the implementation process (The process domain)*		
*CFIR constructs*	*Facilitators*	*Barriers*
Champions	Value of engaged clinic co-workers to advance digital services	

*Indicates mixed or contradicting views among health care providers in different clinics

*(*Table 2. *Perceived barriers and facilitators related to implementation of digital health care services in youth clinics, presented by Consolidated Framework for Implementation Research (CFIR) domains and constructs)*

Below, we present the main findings for each theme, illustrated by quotes from the respondents.

### Digital health services: increased flexibility for the organization, midwives and youth

#### Relative advantages of digital services for youth, for midwives, and for clinics

Respondents found that the addition of digital services improved the timeliness and youth centeredness of the health services which facilitated the implementation and usage of the digital services. The availability of digital appointments was considered high, and the digital format made adaptation of services to youth needs easy. Examples included the ability to easily involve parents or offer multiple follow-up sessions, maintaining contact if a youth moved or went travelling as well as accommodating booking and referral inquiries.

*“If I have spoken* [on the phone] *to a youth who needs oral contraception, they’ve run out today, then I refer them to the online clinic because I know there is usually available appointments there” -* Respondent 12.

The added work flexibility was emphasized as a benefit for the individual midwife, but also for clinics with limited resources, both in terms of health care personnel and clinical office space. Respondents found that the opportunity to work remotely improved their work-life balance. In some clinics, digital options helped mitigate office space challenges and allowed midwives to adhere to safety regulations for solitary work by scheduling digital appointments instead of in person meetings. Another benefit was the ability to fill up available time slots in one’s schedule by allocating them to digital meetings.

*“At that time, we didn’t have many visits at the clinic and it’s so easy to change in our schedules so we could quickly add online appointments. Let’s say that I have a free time slot between 9 and 10 in the morning, then I just add two online appointments and they get booked”–* Respondent 1.

#### Low degree of complexity and appropriateness of design and packaging

The digital software was mainly considered easy to use. Features such as incorporated health forms and digital information packages were highly appreciated. While some found the time frame for digital appointments sufficient, several remarks were made about digital meetings being short and stressful compared to on-site appointments, which was considered a barrier for implementation and usage of digital healthcare. Shortage of time during digital meetings was foremost related to technical issues, such as handling different digital systems simultaneously, but also midwives believes that youth who sought digital care expected short and effective meetings. Additionally, midwives requested clear guidelines regarding what to document in the medical records in digital meetings compared to on-site meetings, i.e. midwives proposed a less extensive medical anamnesis in digital meeting which would decrease the perceived stressfulness.

### The health care system: lowering the threshold for some youth, but efforts needed to ensure equitable access and utilization of services

#### Accommodating to patient needs and resources, but with persisting access inequities

The digital services were described as attentive and appropriate to the needs and preference of youth, which had facilitated the implementation process. Furthermore, digital services removed some known barriers to utilization, such as travel time to and from the clinic. Respondents also believed that digital services lowered the threshold for youth to access the youth clinics, i.e. many digital attendants were first-time visitors or individuals with a history of missing scheduled on-site and follow-up meetings.

*“It’s a great tool for the first contact* [with the youth clinic], *to reach out to us and see that we exist and that we’re not so intimidating or such”–* Respondent 11.

The importance of digital options was emphasized for otherwise hard-to-reach groups such as youth with neuropsychiatric disorders or social anxiety, boys and young men, and youth experiencing stigma or shame related to their sexuality. Some respondents feared that youth living in controlled environments, or a culture of honor would be more vulnerable to digital service inequities. Identified accessibility challenges for youth included the absence of a safe and calm home environment and the lack of electronic identification or required technology.

*“During the pandemic we’ve been urged to have as many digital meetings as possible, also for youth in our neighborhood* [low socioeconomic area], *but it’s been difficult here. Many don’t have the option, they don’t have electronic identification, they live in crowded houses and can’t talk with us undisturbed in their houses”–* Respondent 2.

Digital visitors were described to have a high health literacy. Some respondents pointed out that insufficient effort had been made to engage and inform youth, especially in low socioeconomic- and remote areas where youth had not yet discovered the digital options, which was considered a barrier during the implementation process. Additionally, some respondents found the digital format to be challenging when providing services to younger adolescents (< 15 years), youth with poor language skills, or youth struggling with multiple health conditions or risk behaviors. Concerns that the digital format could exacerbate communication barriers led to midwives’ preferences of meeting these groups in person at the on-site clinics.

#### Peer-pressure from other health care actors and external incentives to scale up digital care alternatives

Emerging digital health actors in the private sector– i.e. competing services from digital health care providers– were considered an important incentive for the implementation of digital services in youth clinics. Respondents emphasized the need to ensure youth did not seek out private caregivers instead of choosing the youth-tailored and free services provided by youth clinics.

*“We have timely services, we’re available digitally, you can have the appointment from the school bathroom or while waiting for the bus, but we’re still a youth clinic. We’re not just another online health actor and hopefully youth feel they can ask more questions here than they would to other care givers”.–* Respondent 9.

### The organization at the youth clinics: mixed experiences of organizational support and digital implications on quality of care

#### Structural characteristics negatively affected by divided management

The implementation of digital health services benefited from– and strengthened– the collaboration between different on-site youth clinics in the region. Some midwives found that the implementation of digital care contributed to standardized practices and organizational cohesiveness, having a positive influence on the overall services offered by the clinics. The divided management structure for medical staff and social counsellors was however highlighted as a limitation, as only midwives were included in the provision of digital services. Many respondents believed this undermined the holistic approach of the youth clinics and hence, reduced the advantages of the digital services and was considered a barrier for usage.

#### Open communication climate within organization but lacking communication on goals and feedback

Generally, midwives described the organizational communication structure to be open and non-hierarchal, but a few respondents expressed that feedback on the digitalization process was insufficiently addressed. The goal of implementing digital services was communicated in a broad manner, mainly stating the need to improve service accessibility. Respondents expressed they would have benefitted from detailed clarification regarding objectives of implementing the digital services. Concerns about the future of on-site clinics and in-person meetings were raised by several respondents who feared digital services might replace rather than complement on-site services.

*“I think we should hold on to digital services, it should exist, but not take over. That’s a concern rather, that we need to keep our youth in on-site appointments. Digital services are only a complement, but a good one”.–* Respondent 7.

#### The importance of compatibility of digital health care services with individual and organizational values, norms and work structures

Midwives found the digital format to be a good complement to on-site services, appropriate for quick and easy inquiries from youth, and often ideal for follow-up with youth after an on-site meeting. A general need for youth clinics to keep up with digitalization and to align with the overall societal change was also expressed. However, many respondents did not find the digital format appropriate for asking youth sensitive questions, and often found it challenging to talk with youth about their psychosocial wellbeing or capturing the “whole person” in the digital meeting, an implementation barrier. This was linked to interpersonal factors, such as the loss of non-verbal cues and body language, but also to patient safety and privacy concerns. In comparison to on-site appointments, midwives had little control over the online meeting environment, such as knowledge of who might overhear the conversation.

*”They contact us regarding a specific problem and then I find there’s not much room for the circumstantial aspects. Maybe they’re on a bus and then I feel like it’s not the right time to ask about sensitive things like exposure to violence”.–* Respondent 12.

Patient safety was on the other hand considered strengthened by the electronic identification requirement and by the ability to contact youth through a safe and confidential chat function. While a few examples of added workload due to the chat were mentioned, most respondents found both video meetings and the chat to fit within or improve their overall work routines, which facilitated the implementation and usage of the digital services.

*“We use chat extensively because it’s such a good tool. I tell everyone attending the clinic to look there for test results or follow-up, or to reach out in the chat if they have any questions”–* Respondent 16.

#### Available resources for implementing the intervention and sufficient access to knowledge and information

Technological infrastructure was sufficient in the youth clinics and real-time IT-support was mostly available and a highly appreciated resource. Most respondents found they had sufficient guidelines and information to use the digital tools, but remarks were also made regarding lack of initial training and demand for further educational opportunities, which had impeded the implementation process. Midwives in some of the smaller clinics also found they had insufficient time for digital appointments, due to the high demand for on-site appointments.

### The health care providers: midwives appreciate the digital option but prefer meeting youth face to face

#### Midwives’ knowledge and beliefs about the intervention over time

Midwives mostly expressed positive attitudes towards digital meetings, viewing it as an exciting new work task. Many respondents, however, stated that they preferred meeting youth in person and therefore booked on-site meetings by default without offering a digital alternative. Respondents did however recognize digital meetings as a convenient alternative for youth and some stated the intent to suggest digital follow-up meetings more often.

*“I really don’t mind digital follow-up meetings, on the contrary, but I don’t think about it in the moment. Sometimes I realize later that this youth doesn’t need to come back all this way, and then I call to change the follow-up to digital format”.–* Respondent 15.

#### Midwives’ self-efficacy in the digital meeting and the importance of prior youth clinic experience

Engaging in digital service delivery was seen as important to gain confidence and skills, but many respondents still felt constrained in digital meetings. This was linked to challenges such as difficulties in building trust with youth, finding conversations to be stressed and stiff, or uncertainty in how to manage emotional reactions from youth in the digital format. Perceived youth expectations on digital health care as a ”fast and easy” alternative also had implications on midwives´ willingness to raise additional questions in the digital meeting. Inability to ask colleagues for help or to conduct physical exams as well as limited access to information material available in the clinic, were other factors affecting confidence when working digitally. For this reason, sufficient experience of working in the youth clinic prior to engaging in digital meetings was stressed as a facilitator by some respondents.

*“When we ask about some things, they can become very emotional […] and you need to consider what kind of reactions you could get from different questions. If I’m sitting in a video meeting I can’t give them a comforting hand, you know what I mean? Here* [at the clinic], *you are more present in the moment, and you can support this person better.”–* Respondent 8.

### Implementation strategies of the digital health services: the importance of knowledgeable and enthusiastic colleagues during the implementation process

#### Positive impact of engaged champions in the clinic

Some of the respondents stated that they had been, or had a colleague who had been, highly engaged in implementing digital services in their clinic. Responses indicated that enthusiastic and knowledgeable colleagues had a positive impact on midwives’ overall perception of the implementing digital services.

*“Since the two of us were in this from the beginning I think we managed to get the others on board, that they found it natural […] and they could see it worked. There’s always a resistance to new things but I think we got the whole clinic with us in this”.– Respondent 16*.

## Discussion

This interview study explored barriers and facilitators to the implementation of digital health care services from the perspective of midwives working in Swedish youth clinics, by using the CFIR framework. Overall, adding digital health care services within youth clinics was positively received by the midwives. The digital format was considered highly useful especially for shorter inquiries and provided a low threshold entry point for youth to access the clinic. Added flexibility, improved timeliness of appointments, and accommodating youth needs strongly influenced the acceptability and use of the digital health care services in a positive direction. However, midwives found it difficult to reach the same conversational depth with youth in digital consultations compared to on-site visits due to communication barriers, privacy and safety concerns, time constraints, and the inability to offer digital appointments for social counselling. These factors negatively affected the implementation and use of digital health care services in youth clinics. Similar to findings across different health care settings,^35^ we were not able to identify a single key facilitator or barrier. Hence, success or failure of implementation of digital health care services was affected by multiple factors that need to be strengthened or addressed.

The needs and resources of the youth population stand out as central for midwives’ acceptability of the digital services. The positive influence of recognized youth benefit such as equality, accessibility, reduced travel time, youth-centered services, and patient-provider continuity, resonates with experiences from other health fields.^21^ Additionally, midwives in our study suggested that different groups within the youth population had found their way to the youth clinics due to the digitalization, e.g. boys and young men and people with neuropsychiatric diagnosis, which was also considered an incentive for the implementation of digital health care services. The organizational objective of youth clinics is to make the services accessible and attractive to promote utilization, therefore, this aspect likely has a strong explanatory power for how digital services were perceived by the respondents, and how facilitators and barriers were discussed. It is from this perspective not surprising that compatibility with organizational values, objectives and workflows stands out as essential for a successful implementation.

A major concern, expressed in this study, was how well the digital format corresponded with the overall objective of the youth clinic, i.e. a holistic approach to youth development. This was foremost highlighted in relation to social counsellors not being involved in digital health care delivery which decreased cross-professional collaboration and affected the ability to support and meet youth needs. In accordance, lack of coordination and collaboration between health care professionals has previously been identified as a barrier to adoption to digital health care tools [17].

Another major barrier to digital health care identified in our study was the interpersonal challenges including loss of non-verbal cues, building trustworthy relations with youth, and difficulties discussing sensitive topics in the digital meeting, which also reflect previous research findings [17, 19–21]. Hence, all types of appointments did not benefit from being held online and on-site follow-up was often mandated. Midwives in our study regarded digital health care services as a complement rather than a “silver bullet” to reach all youth. Researchers have warned against digitalization being seen as a “one size fits all” solution, highlighting the importance of maintaining a patient-centered care [35, 36]. According to the inverse digital care law, digital health may contribute to, or amplify existing social disparities if it is allowed to expand on behalf of on-site access for vulnerable populations [37]. Similarly, midwives in our study expressed a fear of digital solutions overriding on-site visits. In a recent Canadian study, adolescents reflected on digitalization having resulted in decreased on-site accessibility following COVID-19, expressing concerns with increasing waiting-times for SRH services as compared to before the pandemic [38]. In our study, some midwives identified digital health care as an accessibility barrier, affecting youth disproportionately. For example, midwives working in low socioeconomic areas with high proportions of migrant populations, highlighted the accessibility inequities for youth lacking digital identification and living in overcrowded houses. Maintaining on-site accessibility for vulnerable groups was therefore stressed as utterly important.

Previous studies have identified safety concerns in digital health care services both from a health care provider and a youth perspective, either due to insecurities surrounding confidentiality or lack of privacy in the meeting [7, 9, 20, 36, 39]. The primary safety concerns for midwives in our study were the risk of meetings being overheard by a third person, and not having full control over the meeting environment. By contrast, a systematic review by Rea et al. (2022) [7], showed that adolescents largely perceive digital sexual and reproductive health care services to be more private than face-to-face meetings given their anonymity. It is worth noting that among youth, privacy-concerns in digital sexual and reproductive health care have primarily focused on a fear of family/parents eavesdropping or reading notifications on a device, rather than direct conversational control [7, 20]. Youth digital preferences therefore unsurprisingly include anonymous mobile app-icons, as well as a trustworthy and “safe” sender of information, and assurances of confidentiality [7, 38, 40]. According to midwives in our study, the electronic identification protected chat, included in the digital services, provided a safe way to contact and share information with youth. The success of the digital health care service can thus likely be attributed to the acceptability of certain privacy measures from both a health care provider and youth perspective.

Our findings support the overall importance of the health care organization being prepared for the implementation of digital health care services, which was also highlighted in a recent CFIR-based scoping review on telehealth service implementation initiatives [41]. Furthermore, Rangachari et al. (2022) [41] emphasize the importance of leadership engagement for an effective implementation of digital interventions. Similarly, our study found education, training, and organizational support to be desirable and important. As digital health care providers from various settings continue to emphasize the importance of these aspects, learning activities, practical training and support are important factors to consider throughout future implementation processes [39, 41, 42].

Findings from our study and previous literature [21, 35], suggest that defining the best practice for digital health care might be the way forward. Midwives in our study stated they preferred meeting youth in person and were therefore more inclined to make on-site appointments. To overcome this hindering factor, a flowchart or a triage system could help midwives and other health care providers to identify the most suitable meeting form (digital or on-site) for various inquiries. The digital meeting itself could also be seen as a first triage for the youth health inquires. For example, digital contraceptive counselling has previously been identified as acceptable for both health care providers and patients [40]. Other possible matches for digital meetings are renewal of contraceptive prescription, giving out test results, follow up meeting regarding treatment or side effects of treatment.

### Strengths and limitations of the study

Using CFIR to guide data collection and analysis was both a strength and a limitation of this study. The framework offers a greater degree of transparency in the analytical process and strengthens the transferability of the study findings. It also provides a shared language assisting in the interpretation and comparison of results across studies using the same framework [43]. While CFIR provides a comprehensive structure to identify and organize implementation determinants it does however not specify how constructs are linked and interact– other ways of organizing the findings might therefore have conveyed such relationships better.

The late implementation stage at which this evaluation was conducted can be regarded as a limitation. Findings may however still inform adjustments and strategies of the digital services that can be used to further implement and support its sustained use [32, 43]. Ideally research on implementation determinants should be explored prior to implementing the intervention rather than after. The inclusion of other key stakeholders, such as youth clinic management or clinic attendants/youth, would likely have provided additional and more nuanced findings but was not feasible in this study. On the other hand, the results presented here illustrate how interventions (e.g. implementing digital services) are typically carried out in a health care organization setting. The initial stages of such intervention processes– including decision-making and overall strategy– often do not involve the health care providers that will be instrumental in the implementation of said process. In this respect, the study contributes valuable “real life” insights for future research and implementation.

We set out to explore midwives’ perceptions of digital services, however, the recruitment of respondents representing youth clinics in different socioeconomic settings has likely affected the findings, which can be considered a limitation. If the study had exclusively involved midwives working in migrant dense areas, the findings would most probably predominantly be focused on negative aspects of digital services such as accessibility barriers.

One of the studies strengths was the use of two interview periods, as this allowed the study to capture the experiences of midwives at two points of the implementation. During the first interview occasion, public health restrictions due to Covid-19 were still in place and the chat function had just been introduced in most clinics. Thus, the second occasion provided valuable insight into both temporal and persisting challenges and enabled a post-pandemic perspective. Previous reviews have also recommended that future studies should identify the unique perspectives of different groups of healthcare practitioners [39, 42, 44], an additional strength of this study is therefore the focuses on midwives involved in youth SRHR-provision.

## Conclusions

This study contributes to a growing body of implementation research into health care providers perceptions of introducing digital heath care services. Midwives welcomed the option to provide digital sexual and reproductive health care to youth, while still recognizing its limitations. The digital consultations were considered valuable by complementing the on-site encounters with timely, and low-threshold options for youth. However, communication barriers and concerns regarding patient privacy and safety impacted the holistic approach of youth clinics. Additionally, the importance of sufficient training and organizational support to provide high quality digital sexual and reproductive health care services was emphasized. It reiterates the importance of health care providers’ perceptions about how well the new digital services address the needs of youth. Further research is needed to evaluate the effectiveness of digital health care services in meeting unmet sexual and reproductive health care needs, developing guidelines for best practice, and exploration of how young people perceive the services. Knowledge on how and by whom digital health care services are utilized is crucial to ensure services are accessible and equitable when remote health care is increasing. This knowledge may in turn further increase health care providers’ acceptability and adoption of digital services.

### Electronic supplementary material

Below is the link to the electronic supplementary material.

Supplementary Material 1

Supplementary Material 2

## Data Availability

The datasets generated and analyzed during the current study are available from the corresponding author on reasonable request.

## Abbreviations

CFIR: The consolidated framework for implementation research
SRHR: Sexual and reproductive health and rights
WHO: The world health organization
