# Supplementary material for: Implementing digital sexual and reproductive health care services in youth clinics: a qualitative study on perceived barriers and facilitators among midwives in Stockholm, Sweden
Source: BMC Health Serv Res. 2024 Apr 2;24:411. doi: 10.1186/s12913-024-10932-1 (PMC10988956; doi:10.1186/s12913-024-10932-1)
Supplement: Supplementary file 2 — Supplementary Material 2 [file 12913_2024_10932_MOESM2_ESM.docx]

INTERVIEW GUIDE: Midwives' perception of digitization of SRH at youth clinics in the Stockholm

| Overarching theme | Questions | Notes |
| --- | --- | --- |
| Preliminary questions | Can you tell us a little about yourself and your experience working as a midwife?   - How long have you been working at the youth clinic? - How long at this clinic? - What is it like to work with SRHR among young people? |  |
| Digital SRHR care | What are your experiences of working with digital SRH care?   - What is your experience working with digital care options? - In what way and to what extent are you involved in digital healthcare? Since how long? |  |
|  | How can digital healthcare meetings complement on-site meetings?  Explore:   - Contraceptive and unwanted contraception? - Abortion counselling? - Physical problems e.g., discharge, fungus, tight foreskin? - Psychosocial problems e.g., relationships, mental illness? - STI prevention? - Teaching and other assignments?   When does the digital format work well/less well? |  |
|  | How does the use of digital care differ between midwives at the clinic?   - Are there different preferences for the meeting format and what are they based on? - Are you basing your work on any guidelines? Good/bad? |  |
|  | How does the format (digital or in person) affect the interaction between you and the young person?   - For example, do you usually bring up questions with the young person that they did not initially seek help for? Why/why not? - What subjects do you feel comfortable bringing up? |  |
|  | Is there any variation in who you meet in digital care meetings compared to meetings in the clinic?   - Different availability for different groups? Which? - What can be done to achieve digital accessibility? - What do you think digital drop-in could bring to the table? |  |
| Experience of working digitally | How has the organization worked to support midwives in the adaptation to digital care?   - How is digital literacy ensured? Educational opportunities? - How is the quantity and quality of available support perceived? Anything missing? - Technical support and infrastructure? - Is it possible to submit comments/suggest changes? How? To whom? |  |
|  | How do you feel that digital meetings fit in with your other tasks?   - Is there anything that has become easier/harder? - What could be improved? - Perceived flexibility, e.g., by working from home? |  |
| Opportunities for improvement and risk management in digital SRH care | What are the risks with digitalization of SRH healthcare on content and quality?   - How can these risks be minimized and addressed?   Explore:   - Contraception (e.g., prescribing LARC) and unwanted pregnancy? - Abortion counselling? - Physical problems - Psychosocial problems - STI Prevention |  |
|  | How could / should digital SRH care at the youth clinic, developed in the future?   - Aspects to add, change and preserve? - What lessons should we learn from the use of digital tools during the pandemic? |  |
| Conclusion | Is there anything else connected to digital sexual and reproductive health that you would like to add? |  |
|  | Is it okay if I contact you again in case I need to ask you any additional questions? |  |
|  | Age? |  |
